# HDAC9‐mediated calmodulin deacetylation induces memory impairment in Alzheimer's disease

**DOI:** 10.1111/cns.14573

**Published:** 2024-02-07

**Authors:** Hai‐Long Zhang, Shufen Hu, Pin Yang, Han‐Chun Long, Quan‐Hong Ma, Dong‐Min Yin, Guang‐Yin Xu

**Affiliations:** ^1^ Jiangsu Key Laboratory of Neuropsychiatric Diseases, Institute of Neuroscience Suzhou Medical College of Soochow University, Medical Center of Soochow University Suzhou China; ^2^ Key Laboratory of Brain Functional Genomics, Ministry of Education and Shanghai, School of Life Science East China Normal University Shanghai China; ^3^ Department of Neurology The Affiliated Xingyi City Hospital of Guizhou Medical University Xingyi China

**Keywords:** acetylation, Alzheimer's disease, CaM, HDAC9, memory impairment

## Abstract

**Aims:**

Alzheimer's disease (AD) is a neurodegenerative disease characterized by progressive cognitive dysfunction and memory impairment. AD pathology involves protein acetylation. Previous studies have mainly focused on histone acetylation in AD, however, the roles of nonhistone acetylation in AD are less explored.

**Methods:**

The protein acetylation and expression levels were detected by western blotting and co‐immunoprecipitation. The stoichiometry of acetylation was measured by home‐made and site‐specific antibodies against acetylated‐CaM (Ac‐CaM) at K22, K95, and K116. Hippocampus‐dependent learning and memory were evaluated by using the Morris water maze, novel object recognition, and contextual fear conditioning tests.

**Results:**

We showed that calmodulin (CaM) acetylation is reduced in plasma of AD patients and mice. CaM acetylation and its target Ca^2+^/CaM‐dependent kinase II α (CaMKIIα) activity were severely impaired in AD mouse brain. The stoichiometry showed that Ac‐K22, K95‐CaM acetylation were decreased in AD patients and mice. Moreover, we screened and identified that lysine deacetylase 9 (HDAC9) was the main deacetylase for CaM. In addition, HDAC9 inhibition increased CaM acetylation and CaMKIIα activity, and hippocampus‐dependent memory in AD mice.

**Conclusions:**

HDAC9‐mediated CaM deacetylation induces memory impairment in AD, HDAC9, or CaM acetylation may become potential therapeutic targets for AD.

## Graphical abstract

HDAC9 is the CaM deacetylase, which is upregulated in APP/PS1 mouse forebrain neurons, causing CaM hyperdeacetylation. The latter impairs CaMKIIα activity and memory in APP/PS1 mice. HDAC9 inhibition enhances CaMKIIα activity via increasing CaM acetylation, thus improving the memory deficits of APP/PS1 mice.

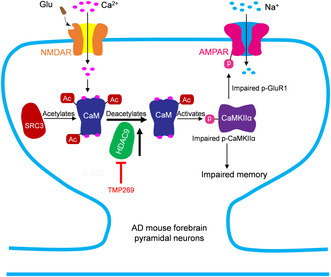

## 1 INTRODUCTION

Alzheimer's disease (AD) is a progressive neurodegenerative disorder, clinically manifested with memory impairment and cognitive decline.^1^, ^2^, ^3^ Abnormal post‐translational modification (PTM), such as protein acetylation, is one of essential mechanisms contributing to AD pathology. While protein acetylation has been implicated in AD before, this was mainly for histone acetylation.^4^, ^5^, ^6^, ^7^, ^8^ Nonhistone acetylation has been shown to regulate extensive biological processes such as DNA repair, cytoskeleton dynamics, cell metabolism, autophagy, and memory.^9^, ^10^, ^11^, ^12^, ^13^, ^14^, ^15^ However, the roles of nonhistone acetylation in memory impairment of AD are less well understood.

Acetylation of lysine at the ε‐amino group is regulated by lysine acetyltransferase (KAT) and lysine deacetylase (HDAC).^16^ The previous focus is that tau acetylation can competitively inhibit tau ubiquitination and degradation, and leading to tau accumulation and neurofibrillary tangles.^17^, ^18^, ^19^, ^20^ However, how nonhistone acetylation abnormalities cause memory impairment in AD are less explored.

Calcium signaling is essential for learning and memory, and is activated by ion channels such as *N*‐methyl‐D‐aspartic acid (NMDA) receptor and voltage‐dependent Ca^2+^ channel (VDCC). Calcium signaling is mediated through several Ca^2+^‐binding proteins like CaM. CaM is a ubiquitous Ca^2+^ sensor and has more than 300 target proteins including protein kinases, enzymes, cytoskeleton proteins, and ion and water channels.^21^, ^22^, ^23^, ^24^, ^25^, ^26^ For instance, CaM interacts with and activates CaMKIIα, which is important for several functions including learning and memory in the brain.^26^, ^27^, ^28^

Here, we found that CaM acetylation was reduced in AD patient and mouse plasma. We further showed that CaM acetylation and its target CaMKIIα activity were severely impaired in AD mouse brain. We performed an unbiased screen and identified HDAC9 as the main deacetylase for CaM. HDAC9 inhibition increased CaM acetylation and CaMKIIα activity, and hippocampus‐dependent memory in AD mice. Together, these results provided evidence for a role of HDAC9‐mediated CaM deacetylation in memory impairment of AD, and revealed dynamic CaM acetylation in the pathogenesis of AD. The study indicated HDAC9 or CaM acetylation may become new drug targets for AD treatment.

## 2 MATERIALS AND METHODS

### 2.1 Animals

C57BL/6J and APP/PS1‐transgene male mice at age of 7 months were used in experiments unless otherwise described. Animals were housed in rooms at 23°C in a 12/12 h light/dark cycle and with food and water available ad libitum. All experimental procedures were approved by the Institutional Animal Care and Use Committee of the Soochow University (SYXK 2022‐0043). All animal experiments followed the ARRIVE guidelines.

### 2.2 Human blood samples

Ten AD patients (6 male, 4 female; 69.9 ± 9.5 years old) diagnosed as previously described,^29^ were recruited from the Affiliated Xingyi City Hospital of Guizhou Medical University. A diagnosis of MCI included: (1) memory complaint; (2) clinical dementia rating 0.5; and (3) normal general cognitive function and daily life activities. A diagnosis of AD included: (1) the NINCDS‐ADRDA criteria (the National Institute of Neurological and Communicative Disorders and Stroke‐Alzheimer's Disease and Related Disorders Association); (2) Mini‐Mental State Examination score of ≤26; and (3) the exclusion of brain tumor, vascular dementia, and cerebrovascular diseases. Ten healthy elders (4 males, 6 females; 74 ± 7.9 years old) were recruited as a control group for the AD patients. No statistically significant differences in age were found between the AD and corresponding control groups. Plasma was collected for Western blotting and stoichiometry. The Ethics Committee of Xingyi City Hospital approved the study and informed consent was given (Approval code: 202204251136000543809). Human subject information is listed below.

**Table:** 

Group	No.	Gender	Age	Nationality
Non‐AD elders	1	Woman	78	Han
	2	Man	77	Han
	3	Woman	76	Han
	4	Man	72	Han
	5	Man	63	Han
	6	Woman	84	Han
	7	Man	63	Han
	8	Woman	76	Han
	9	Woman	85	Han
	10	Woman	66	Han
AD patients	1	Man	61	Han
	2	Woman	78	Han
	3	Woman	72	Han
	4	Man	54	Han
	5	Man	78	Han
	6	Woman	69	Han
	7	Woman	85	Han
	8	Man	74	Han
	9	Man	60	Han
	10	Man	68	Han

### 2.3 Western blotting

Western blotting was performed as described in previous study.^30^ Homogenates of forebrain or hippocampus tissues were prepared in a RIPA buffer containing 50 mM Tris–HCl, pH 7.4, 150 mM NaCl, 2 mM EDTA, 1% sodium deoxycholate, 1% Triton X‐100, 1 mM PMSF, 50 mM sodium fluoride, 1 mM sodium vanadate, 1 mM DTT, and protease inhibitors cocktails. Total protein solutions were mixed with 5 × SDS‐PAGE sample buffer. All the protein samples were boiled in a 95°C water bath for 10 min before Western blotting. The homogenates were resolved on SDS‐PAGE and transferred to PVDF membranes, which were incubated in the TBS buffer containing 0.1% Tween‐20 and 3% BSA for 1 h at room temperature before incubation with a primary antibody overnight at 4°C. After washing, the membranes were incubated with an HRP‐conjugated secondary antibody in the same TBS buffer for 1 h at room temperature. Immunoreactive bands were visualized by ChemiDoc™ XRS Imaging System (BIO‐RAD) using enhanced chemiluminescence (Pierce) and analyzed with Image J (NIH). The primary antibodies used were as follows: anti‐Calmodulin (CaM) (Millipore, 05‐173), anti‐Acetylated‐Lysine (Cell Signaling, 9441), anti‐CaMKIIα (Cell Signaling, 11945), anti‐p‐CaMKIIα Thr^286^ (Sigma, SAB4300228), anti‐GluR1 (Abcam, ab109450), anti‐p‐GluR1 Ser^831^ (Abcam, ab109464), anti‐PSD95 (Millipore, MAB1596), anti‐SRC3 (Cell Signaling, 5765), anti‐α‐tubulin (Cell Signaling, 3873), anti‐HDAC4 (Cell Signaling, 7628), anti‐HDAC6 (Abcam, ab239362), anti‐HDAC7 (Abcam, ab166911), anti‐HDAC8 (Abcam, ab187139), anti‐HDAC9 (Abcam, ab18970).

### 2.4 Detection of CaM acetylation

Lysates were incubated with acetyllysine antibody‐conjugated agarose (Immune Chem, ICP0388) on a rotor shaker at 4°C overnight. After incubation, agarose beads were washed with PBST 4 times through repeating centrifugation and aspiration. A volume of 60 μL of 2 × SDS‐PAGE loading buffer without DTT or 2‐mercaptoethanol was added to the beads, vortexed and boiled for 5 min. After centrifuging the beads at 1000 *g* for 2 min, the supernatant was used for Western blotting with mouse anti‐CaM antibodies. To generate site‐specific anti‐acetylated CaM antibodies against K22, K95 or K116, rabbits were immunized with 500 μg of peptides C‐FSLFD (Ac‐K) DGDGT, C‐FRVFD (Ac‐K) DGNGY and C‐TNLGE (Ac‐K) LTDEE with complete Freund's adjuvant 3 times, and boosted with 250 μg peptides with incomplete Freund's adjuvant additional 4 times. Rabbit anti‐serum was collected and purified by respective nonacetylated peptides.^31^

### 2.5 Constructs of HDAC shRNA

The HDAC shRNA constructs were kindly provided by Dr. Jef D. Boeke from Johns Hopkins University. The shRNAs were cloned from pLKO.1 vector into the NdeI‐BamHI sites of pLKO.1‐hPGK‐Neo vector (Sigma‐Aldrich). The targeting sequence against human HDAC are as follows:

shHDAC4, CGACTCATCTTGTAGCTTATT;

shHDAC6, CATCCCATCCTGAATATCCTT;

shHDAC7, GCCAGCAAGATCCTCATTGTA;

shHDAC8, GCATTCTTTGATTGAAGCATA; and

shHDAC9, CCTAGAATCTTTGTGAGGTTT.

### 2.6 Cell culture and transient transfection

HEK293 cells were grown at 37°C in Dulbecco's modified Eagle's medium supplemented with 10% fetal bovine serum, 100 U/mL penicillin, and 100 mg/mL streptomycin. HEK293 cells were transfected with different HDAC shRNA plasmids using linear PEI (Polysciences, 23966‐2). The HDAC shRNA plasmids were all Flag‐tagged. The pan HDAC inhibitor TSA is from Sigma (T1952) and was used as a positive control to analyze CaM acetylation.

### 2.7 Purification of site‐specifically acetylated CaM recombinant proteins

The site‐specifically acetylated CaM recombinant proteins were synthesized according to a previous report.^32^ In brief, Escherichia coli strain, BL21 (DE3), was transformed with plasmids pAcKRS‐3 and pCDF PylT‐1 carrying the ORF for CaM with an amber codon at the desired site. The cells were first grown overnight in LB medium supplemented with 50 mg/mL kanamycin and 50 mg/mL spectinomycin (LB‐KS) at 37°C. Two mL of bacteria were cultured overnight and then inoculated into 200 mL LB‐KS for further culturing. When the OD600 reached 0.4 ~ 0.6, 20 mM nicotinamide (NAM) and 10 mM acetyl‐lysine were added and 30 min later, the protein expression was induced at 18°C overnight by adding 0.5 mM IPTG. Cells were harvested after induction, and were washed with ice‐cold PBS containing 20 mM NAM, the proteins were purified with HisTrap FF (GE Healthcare, 17‐5319‐01) according to the manufacturer's protocol.

### 2.8 In vitro deacetylation assay

Deacetylation was assayed as previously described.^33^ In brief, Flag‐HDAC9 was overexpressed in HEK293 cells and purified with anti‐Flag M2 affinity gel by following vendor's instructions. Purified Flag‐HDAC9 was resolved on SDS‐PAGE and stained by coomassie blue to verify the amount and purity. Purified His‐CaM (1 μg) was first acetylated by SRC3 (1 μg) in vitro, then acetylated His‐CaM was incubated with or without Flag‐HDAC9 (1 μg) in the 1 × deacetylation buffer (20 mM Tris [pH 8.0], 150 mM NaCl, and 10% glycerol) at 30°C for 1 h. The reaction was stopped by adding 5 × SDS‐PAGE sample buffer and boiled in 95°C for 10 min.

### 2.9 Stoichiometry of CaM acetylation at K22, K95, and K116

We used purified acetylated CaM proteins as standards to analyze the stoichiometry levels for each acetylated lysine residue of CaM under healthy and AD conditions. The standard samples containing 0%, 0.1%, 0.3%, 1%, 3%, and 10% acetylated His‐CaM proteins in 1 μg total His‐CaM proteins were subjected to ELISA assay in 96‐well microplates which were coated with 100 μL of site‐specific anti‐Ac‐CaM antibodies (0.01 μg/mL) overnight. The forebrain lysates with 100 μg proteins were used for the same ELISA assay considering that the endogenous CaM proteins were about 1% of total proteins in the forebrain.^34^ The standard samples and forebrain lysates with the volume of 100 μL was added to each well and was incubated for 2 hours at room temperature, and then each well was washed with PBS containing 0.1% Tween‐20 for 3 times. After washing, 100 μL of secondary antibodies (HRP‐conjugated goat‐anti‐rabbit IgG, 1 μg/mL) was added to each well and incubated for 2 h at room temperature. After that, each well was washed with PBS containing 0.1% Tween‐20 for 3 times before adding 100 μL HRP substrate solution (1:1 mixture of color reagent A H_2_O_2_ and color reagent B tetramethylbenzidine, R&D Systems, DY999) into each well. After incubation with the HRP substrate solution for 20 min at room temperature, 50 μL of stop solution (2 N H_2_SO_4_, R&D Systems, DY994) was applied to each well. The optical density of each well was determined using a microplate reader set to 450 nm. We first generated a standard curve for stoichiometry of standard samples containing different concentration of Ac‐CaM, and then determined the stoichiometry levels of endogenous Ac‐CaM proteins in the forebrain lysates.

### 2.10 Administration of TMP269 into lateral ventricles

A permanent cannula was placed in the right lateral ventricle with the coordinates: AP −0.58 mm, ML +1.20 mm, DV −2.00 mm relative to bregma. Animals were allowed to recover from surgery for a week before experiments. The infusion cannula was connected via PE20 tubing to a microsyringe driven by a microinfusion pump (KDS 310, KD Scientific). The HDAC9 inhibitor TMP269 (TOCRIS) was prepared in aCSF (0.1 M) and 5 μL stocking solution was injected into the right lateral ventricle through infusion cannula. The 7‐month‐old APP/PS1 mice were injected with TMP269 into the lateral ventricle. Half an hour after injection, APP/PS1 mice were trained to perform molecular and behavioral tests. The injection sites were examined at the end of the experiments, and animals with incorrect injection site were excluded from the data analysis.

### 2.11 Contextual fear conditioning

The investigators who performed behavioral analysis were blinded to the treatment of the mice. Seven‐month‐old mice were first habituated to the behavioral room and apparatus (Fear Conditioning System, Panlab) for 5 min. During training, mice were placed in the conditioning chamber and exposed to three foot shocks (2 s, 0.5 mA) with an interval of 30 s. One day after training, mice were returned to the chamber to evaluate contextual fear memory. Freezing during training and testing was scored using PACKWIN software. Data were expressed as percent freezing in 180 s epochs, with each epoch divided into 12 bins.

### 2.12 Morris water maze

The Morris water maze (MWM) consists of a circular pool (diameter = 120 cm, height = 50 cm) filled with water maintained at room temperature (25 ± 1°C) and made opaque with nontoxic white paint. Mice were trained for 5 days with 4 trials per day and 120 s per trial. We used four extra‐maze visual cues to ensure that visual spatial memory was used by the mouse to find the hidden platform location. Twenty‐four hours after the last trial of training (day 6), the platform was removed and all mice were given one trial for 60 s searching (probe test).

### 2.13 Novel object recognition

For the habituation phase, individual adult male mice were placed in a chamber (50 × 50 × 40 cm) and allowed to freely explore the context for 10 min, while being recorded by an overhead camera. For the sampling phase, the animal was placed in the same chamber containing two different objects for 5 min and allowed to explore the object. To saturate the odor left by the previous mice and to make the mice more relaxed, we placed standard animal beddings in the chamber. For the testing phase, one of the objects was exchanged with a new one, and the time spent exploring the two objects was separately recorded using ANY‐MAZE software. The discrimination index (DI) was calculated using the following formula: (time exploring the novel object − time exploring the familiar object)/(time exploring the novel object + time exploring the familiar object) × 100.

### 2.14 Statistical analyses

All data are presented as mean values ± SEM, and normality was checked for all data before comparison. Data with a minimal probability that the animal's value deviates from the mean by >2 SDs are defined as abnormal data. The remaining data are analyzed statistically to obtain the results in the graph. The example data shown were close to the overall mean. Comparisons between two groups were made using unpaired *t* test. Comparisons between three or more groups were made using one‐way ANOVA analysis followed by Tukey's post‐hoc test. The data about learning curve of MWM were analyzed by two‐way ANOVA. Statistically significant difference was indicated as follows: *p < 0.05, **p < 0.01, and ***p < 0.001. The statistical analysis was performed with the software of GraphPad Prism 9.

## 3 RESULTS

### 3.1 Alteration of CaM acetylation in AD patients and mice

**FIGURE 1:**
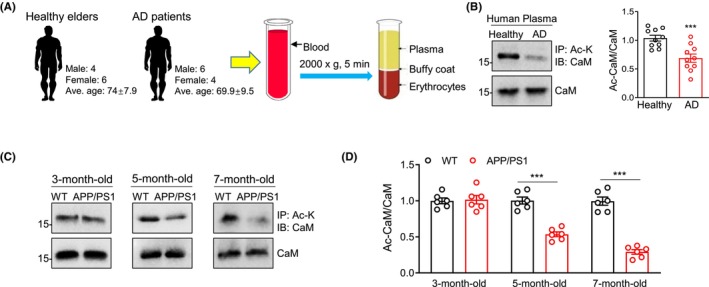
CaM acetylation is reduced in AD patient and mouse plasma. (A) human plasma collection diagram. (B) CaM acetylation in healthy elder and AD patient plasma. The human plasma was immunoprecipitated by antiacetyllysine antibodies and then were immunoblotted with anti‐CaM antibodies. The lower CaM blots were of input samples. Right, quantification of Ac‐CaM/CaM. Data were represented as mean ± SEM. ***p < 0.001, compared to control, unpaired *t*‐test, *n* = 10, data were normalized to control. (C) CaM acetylation in 3‐month‐old, 5‐month‐old, and 7‐month‐old WT and APP/PS1 mouse plasma. The mouse plasma was immunoprecipitated by anti‐acetyllysine antibodies and then were immunoblotted with anti‐CaM antibodies. The lower CaM blots were of input samples. (D) quantification of Ac‐CaM/CaM. Data were represented as mean ± SEM. ***p < 0.001, compared to control, unpaired *t*‐test, *n* = 6, data were normalized to control.

We recruited 10 AD patients (6 males, 4 females; 69.9 ± 9.5 years old) and 10 healthy elders (4 males, 6 females; 74 ± 7.9 years old), and obtained human plasma by blood centrifugation (Figure 1A). The human plasma was immunoprecipitated by anti‐acetyllysine antibodies and then were immunoblotted with anti‐CaM antibodies. Reduced levels of CaM acetylation were observed in the plasma of AD patients, in comparison to that in healthy elders (Figure 1B). APP/PS1 transgenic mice, a mouse model commonly used in AD research, harbor appearance of A‐beta plaques starting at 3 months old of age, exhibiting cognitive deficits at 7 months old of age.^35^, ^36^, ^37^, ^38^, ^39^, ^40^, ^41^ Decreased CaM acetylation was detected in the plasma of 5‐month‐old and 7‐month‐old but not 3‐month‐old APP/PS1 transgenic mice (Figure 1C,D). Considering the fact that APP/PS1 mice do not exhibit obvious memory impairment until 7‐month‐old, this result indicates a causal link of CaM acetylation with cognitive deficits of AD mice. Together, these results demonstrate that CaM acetylation is reduced in AD patient and mouse plasma.

**FIGURE 2:**
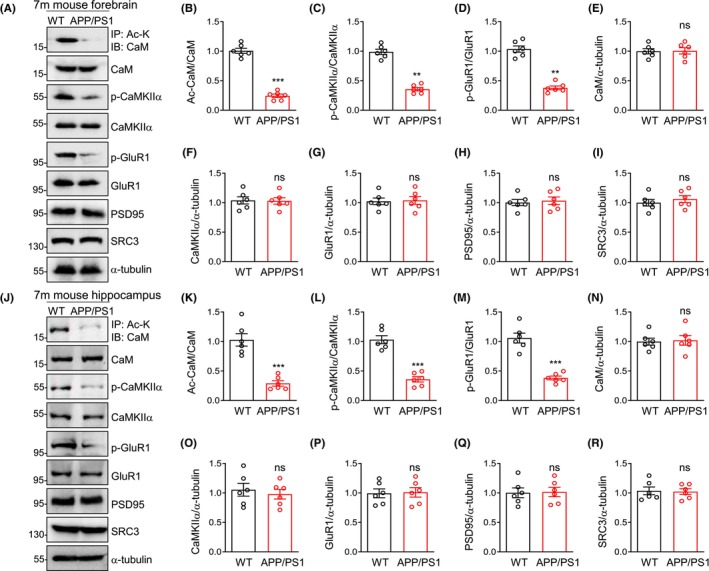
CaM acetylation is decreased in 7‐month‐old AD mouse brain. (A) reduced Ac‐CaM, p‐CaMKIIα and p‐GluR1 in APP/PS1 mouse forebrain. (B–I) quantification of Ac‐CaM/CaM (B), p‐CaMKIIα/CaMKIIα (C), p‐GluR1/GluR1 (D), CaM/α‐tubulin (E), CaMKIIα/α‐tubulin (F), GluR1/α‐tubulin (G), PSD95/α‐tubulin (H), and SRC3/α‐tubulin (I) in panel A. (J) reduced Ac‐CaM, p‐CaMKIIα and p‐GluR1 in APP/PS1 mouse hippocampus. (K–R) quantification of Ac‐CaM/CaM (K), p‐CaMKIIα/CaMKIIα (L), p‐GluR1/GluR1 (M), CaM/α‐tubulin (N), CaMKIIα/α‐tubulin (O), GluR1/α‐tubulin (P), PSD95/α‐tubulin (Q) and SRC3/α‐tubulin (R) in panel J. Data were represented as mean ± SEM. ***p < 0.001, **p < 0.01, unpaired *t*‐test, *n* = 6, data were normalized to WT mice.

Consistent with the plasma results, we used the APP/PS1 transgenic mice to deeply study the pathological mechanism, and found that CaM acetylation was decreased in the forebrain or hippocampus in 7‐month‐old APP/PS1 mice relative to WT mice (Figure 2A,B,J,K). CaM interacts with and activates CaMKIIα which is important for several neuronal functions including learning and memory in the brain.^21^, ^22^, ^23^, ^24^, ^25^, ^26^ Autophosphorylated CaMKIIα was detected with phospho‐specific antibody against Thr^286^, whose phosphorylation is an indicator of CaMKIIα activation.^42^ Consistent with the reduction of CaM acetylation in APP/PS1 mice, p‐CaMKIIα Thr^286^ also decreased in APP/PS1 mice (Figure 2A,C,J,L). CaMKIIα could phosphorylate AMPA receptor subunit GluR1 at Ser^831^ during LTP.^43^, ^44^ Likewise, p‐GluR1 Ser^831^ also decreased in APP/PS1 mice (Figure 2A,D,J,M). While total CaM, CaMKIIα, GluR1, and PSD95 did not change significantly in WT and APP/PS1 mice (Figure 2A,E–H,J,N–Q). In previous studies, we identified that SRC3 was the CaM acetyltransferase, and revealed that CaM acetylation was necessary for CaMKIIα activation.^31^, ^45^, ^46^ We studied whether decreased CaM acetylation is caused by reduced SRC3, and found that SRC3 did not change significantly in APP/PS1 mice, compared to WT mice (Figure 2A,I,J,R). This result perhaps suggests that decreased CaM acetylation may be due to elevated CaM deacetylase in APP/PS1 mice. Altogether, these results demonstrate that CaM acetylation and its target CaMKIIα activity are severely impaired in AD mouse brain.

### 3.2 HDAC9 is the main CaM deacetylase

**FIGURE 3:**
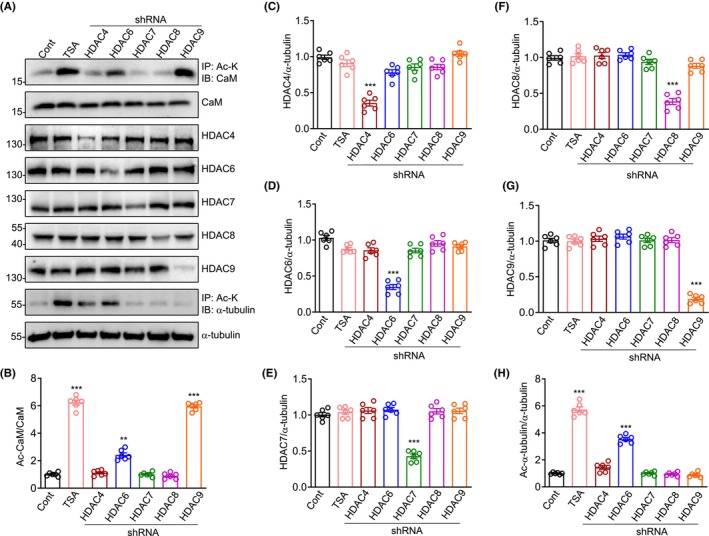
HDAC9 is the main deacetylase for CaM in HEK293 cells. (A) shRNA against HDAC6 and HDAC9 increased Ac‐CaM. TSA, the HDAC inhibitor is used as a positive control. Increased of Ac‐α‐tubulin by HDAC6 shRNA is another positive control. The lysate from HEK293 cells under different treatment or transfection were probed with the indicated Abs. B‐H, quantification of Ac‐CaM/CaM (B), HDAC4/α‐tubulin (C), HDAC6/α‐tubulin (D), HDAC7/α‐tubulin (E), HDAC8/α‐tubulin (F), HDAC9/α‐tubulin (G) and Ac‐α‐tubulin/α‐tubulin (H) in panel A, compared to control. **p < 0.01, ***p < 0.001, one‐way ANOVA followed by Newman–Keuls multiple comparison test, *n* = 6, data were normalized to control and represented as mean ± SEM.

The CaM deacetylase is currently unknown. To screen which HDAC participate in the deacetylation of CaM, we performed shRNA screen against several cytoplasmic HDAC in HEK293 cells. As a positive control, treatment with HDAC inhibitor TSA significantly increased Ac‐CaM (Figure 3A,B). The knockdown efficiency of HDAC shRNAs was verified by Western blotting (Figure 3A,C–G). Consistent with a previous report,^47^ HDAC6 shRNA significantly increased the level of Ac‐α‐tubulin (Figure 3A,H), serving as another positive control. Both HDAC9 and HDAC6 shRNA caused the elevation of Ac‐CaM, while HDAC9 shRNA exhibited stronger capability of enhancing Ac‐CaM than that of HDAC6 shRNA (Figure 3A,B). In contrast, HDAC9 shRNA failed to increase the acetylation of α‐tubulin (Figure 3A,H), suggesting the specificity of HDAC9 in deacetylation of CaM. Altogether these results demonstrate a role of HDAC9 in deacetylation of CaM. We also noted that the level of CaM acetylation might correlate with HDAC knockdown efficiency, and to confirm whether HDAC9 directly deacetylates CaM, we performed the following in vitro deacetylation experiments.

**FIGURE 4:**
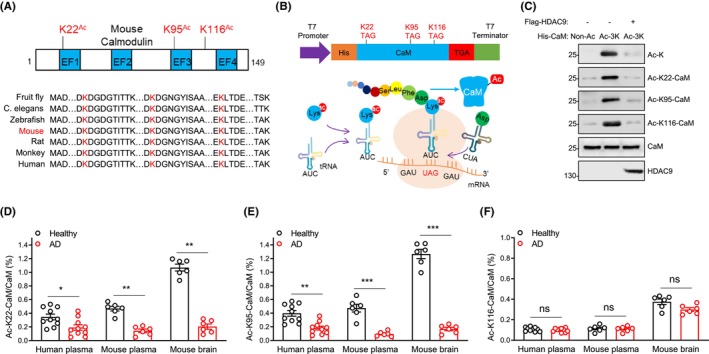
HDAC9 deacetylates acetylated‐CaM and reduced Ac‐K22, K95‐CaM acetylation in AD patients and mice. (A) top, diagram showing the position of acetyllysines in mouse CaM. EF, EF hands. Numbers represent amino acids. Bottom, alignment of the amino acid sequence of CaM from different species. K in red color indicates acetyllysines. (B) top, schematic diagram of pCDF constructs expressing His‐tagged acetylated‐CaM proteins in bacteria. Bottom, diagram showing the strategy of genetic code expansion used to generate site‐specifically acetylated‐CaM proteins. (C) Western blotting to verify expressing His‐tagged acetylated‐CaM proteins in bacteria. HDAC9 deacetylates in vitro acetylated‐CaM at K22, K95 and K116. (D–F) stoichiometry levels of Ac‐K22‐CaM (D), Ac‐K95‐CaM (E) and Ac‐K116‐CaM (F) from human and mouse plasma or forebrain lysates. Data were represented as mean ± SEM, *p < 0.05, **p < 0.01, ***p < 0.001, and two‐way ANOVA followed by Sidak's multiple comparisons test, human plasma samples, *n* = 10; mouse plasma or forebrain samples, and *n* = 6.

We previously reported that CaM is acetylated on three lysine residues (K22, K95 and K116) that are conserved across species^31^ (Figure 4A). To identify whether HDAC9 directly deacetylates CaM, we purified site‐specifically acetylated recombinant CaM proteins using the strategy of genetic code expansion (Figure 4B). This strategy uses an engineered pyrrolysyl‐tRNA synthetase specific for Ac‐K and its cognate tRNA^Pry^ to incorporate Ac‐K at an assigned codon to produce site‐specifically acetylated proteins.^31^ To determine which of the three lysine residues in CaM was acetylated, we generated site‐specific antibodies against Ac‐CaM at K22, K95 and K116.^31^ As shown in Figure 4C, the total Ac‐CaM, Ac‐K22, K95 and K116‐CaM were detected, respectively. The acetylation of CaM at the above lysine residues was reduced by coincubating these purified site‐specifically acetylated His‐Ac‐3K‐CaM, His‐Ac‐K22, K95, and K116‐CaM, respectively, with Flag‐tagged HDAC9 purified from HEK293 cells (Figure 4C), demonstrating that HDAC9 deacetylates CaM directly at K22, K95, and K116 in vitro. These results demonstrate that CaM is a direct substrate of HDAC9.

### 3.3 Stoichiometry of CaM acetylation at K22, 95, and 116 in AD patients and mice

To accurately measure the ratio of Ac‐CaM to total CaM under healthy and AD conditions, we used purified Ac‐CaM proteins as standards to analyze the stoichiometry levels for each lysine residue under healthy and AD conditions. The standard samples containing 0%, 0.1%, 0.3%, 1%, 3%, and 10% acetylated His‐CaM proteins in 1 μg of total His‐CaM proteins were subjected to ELISA assay in 96‐well microplates which were coated with 100 μL site‐specific anti‐Ac‐CaM antibodies (0.01 μg/mL) against Ac‐CaM at K22, K95, and K116.^31^ The optical density of each well was determined using a microplate reader set to 450 nm. We generated a standard curve for stoichiometry of standard samples containing different concentration of Ac‐K22, K95, and K116‐CaM, and then determined the stoichiometry levels of endogenous Ac‐CaM proteins in the lysates. The stoichiometry levels of Ac‐K22, K95, and K116‐CaM in the total lysates were 0.346%, 0.398%, and 0.106% in healthy human plasma, and decreased to 0.191%, 0.181%, and 0.099% in AD human plasma (Figure 4D–F). The stoichiometry levels of Ac‐K22, K95, and K116‐CaM in the total lysates were 0.469%, 0.474%, and 0.114% in 7‐month‐old WT mouse plasma, and reduced to 0.143%, 0.095%, and 0.109% in 7‐month‐old APP/PS1 mouse plasma (Figure 4D–F). The stoichiometry levels of Ac‐K22, K95, K116‐CaM in the total lysates were 1.07%, 1.267%, and 0.375% in 7‐month‐old WT mouse brains, and lowered to 0.205%, 0.168%, and 0.299% in 7‐month‐old APP/PS1 mouse brains (Figure 4D–F). The stoichiometry results showed that the acetylation of CaM at both K95 and K22 were decreased in the plasma and brains of AD patients or transgenic mice, in comparison to that of healthy patients or WT mice, respectively, whereas Ac‐K95‐CaM reduced in a bigger extent, compared to that of Ac‐K22‐CaM. In contrast, Ac‐K116‐CaM exhibited comparable levels in the plasma and brains of either AD patients or transgenic mice, compared to their respective controls. Taken together, these results indicate that Ac‐K22, K95‐CaM declines in both the plasma and brains of AD patients or transgenic mice.

### 3.4 HDAC9 inhibition increased CaM acetylation in the APP/PS1 mouse brain

**FIGURE 5:**
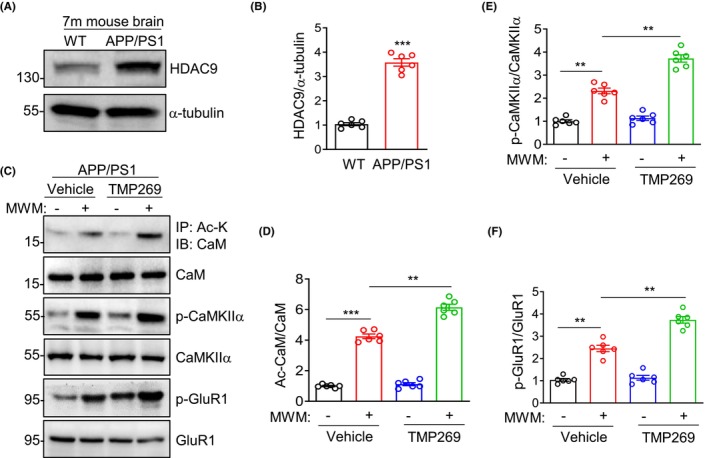
HDAC9 inhibition increases CaM acetylation and CaMKIIα activity during MWM in 7‐month‐old AD mice. (A) representative blots, elevated HDAC9 protein levels in forebrain from 7‐month‐old APP/PS1 mice. (B) quantification of HDAC9/α‐tubulin. ***p < 0.001, unpaired *t*‐test, *n* = 6. Data are presented as mean ± SEM and were normalized to WT mice. (C) increased Ac‐CaM, p‐CaMKIIα and p‐GluR1 after MWM training were enhanced by TMP269 into the lateral ventricle. The total lysates of forebrain after different treatments were probed for Ac‐CaM, CaM, p‐CaMKIIα, CaMKIIα, p‐GluR1 and GluR1. (D–F) quantification of Ac‐CaM/CaM (D), p‐CaMKIIα/CaMKIIα (E) and p‐GluR1/GluR1 (F) in panel C. Data were represented as mean ± SEM. **p < 0.01, ***p < 0.001, one‐way ANOVA followed by Newman–Keuls multiple comparison test, *n* = 6, data were normalized to control.

To investigate whether the decreased CaM acetylation in the brain of APP/PS1 mice is caused by elevated HDAC9, we detected the expression of HDAC9 in 7‐month‐old WT and APP/PS1 mouse brain, and showed that HDAC9 was abnormally increased in APP/PS1 mouse brain (Figure 5A,B). To determine whether HDAC9 is important for CaM deacetylation and CaMKIIα activity in brain, the 7‐month‐old APP/PS1 mice were injected with TMP269, a HDAC9 inhibitor,^48^, ^49^ into the lateral ventricle. Half an hour after injection, APP/PS1 mice were trained to learn to find the hidden platform of the MWM based on visual cues. After the MWM training and learning, the mice were sacrificed for brain CaM acetylation detection. Both the CaM acetylation and the CaMKIIα activation in APP/PS1 brains were enhanced by MWM training, that was further enhanced by HDAC9 inhibition of TMP269 (Figure 5C–E), suggesting a neural activity‐dependent mechanism. CaMKIIα could phosphorylate the AMPA receptor subunit GluR1 at Ser^831^ during learning.^43^, ^44^ As one of substrates of CaMKIIα, p‐GluR1 Ser^831^ exhibited a similar change to both the acetylation and activation of CaMKIIα upon MWM training and TMP269 treatment (Figure 5C,F). The original blots were showed in the Supporting Information (Figures S1–S5). These results indicate that HDAC9 inhibition increases CaM acetylation, accompanied with enhancing CaMKIIα activity in APP/PS1 mouse brain, providing a link between acetylation and activity of CaM.

### 3.5 HDAC9 inhibition improved the hippocampus‐dependent memory in APP/PS1 mice

**FIGURE 6:**
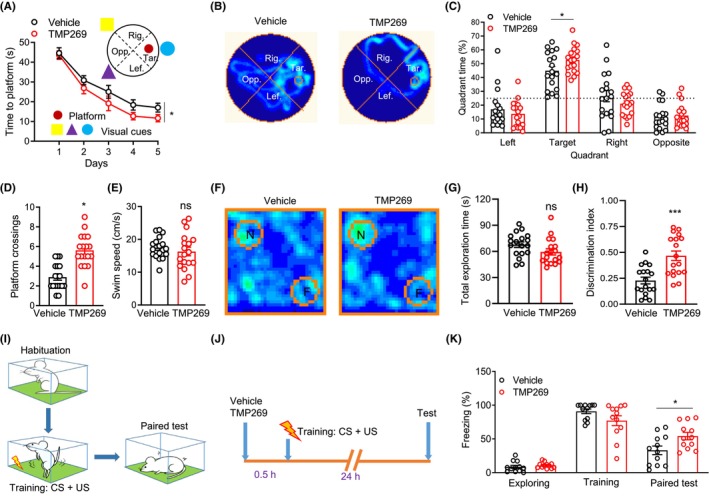
HDAC9 inhibition improves hippocampus‐dependent memory in 7‐month‐old AD mice. (A) learning curve from vehicle and TMP269 group during the training in the water maze. *p < 0.05, *F* (1, 34) = 4.382, two‐way ANOVA, *n* = 18. (B) swimming traces in the water maze during probe test. (C) TMP269 group have increased time staying in the target quadrant, compared to vehicle group. *p < 0.05, two‐way ANOVA followed by Tukey's multiple comparisons test, *n* = 18. (D) increased platform crossings in TMP269 group during probe test, *p < 0.05, unpaired *t*‐test, *n* = 18. (E) similar swimming speed between vehicle and TMP269 group in the water maze. ns, not significant, unpaired *t*‐test, *n* = 18. (F) occupancy plot of the mouse's head point around novel (N) and familiar (F) object from vehicle and TMP269 group. (G) similar total exploration time with N and F between vehicle and TMP269 group. ns, not significant, unpaired *t*‐test, *n* = 18. Data were presented as mean ± SEM. (H) enhanced novel object recognition in TMP269 group. The DI was quantified. ***p < 0.001, unpaired *t*‐test, *n* = 18. Data were presented as mean ± SEM. (I) Schematic diagrams of contextual fear conditioning test. CS, conditioned stimulus (contextual box); US, unconditioned stimulus (foot shock). (J) Schematic diagram of behavioral groups and tests. The 7‐month‐old APP/PS1 mice were injected with vehicle or TMP269 into the lateral ventricle 0.5 h before contextual fear training. (K) Rescued freezing during paired tests by HDAC9 inhibition in APP/PS1 mice. *p < 0.05, two‐way ANOVA followed by Tukey's multiple comparisons test, *n* = 12. Data were presented as mean ± SEM.

Considering the role of HDAC9 on the activity of CaM, which is one of pivotal regulatory factors of hippocampus‐dependent learning and memory,^50^, ^51^, ^52^, ^53^, ^54^ we investigated whether HDAC9 inhibition ameliorates learning and memory impairment in APP/PS1 mice. The APP/PS1 mice injected with TMP269 at 7 months of age were subjected to behavioral tests. In MWM training and learning, the escape latency of TMP269‐treated APP/PS1 mice was decreased, compared to that of the vehicle‐treated APP/PS1 mice (Figure 6A), although the swimming speed was comparable between two groups of mice (Figure 6E). During probe tests, the time that the mice spent in the target area and the number of platform that the mice crossing were increased in TMP269‐treated, compared to vehicle‐treated APP/PS1 mice (Figure 6B–D). These results indicate that HDAC9 inhibition attenuates spatial memory impairment of APP/PS1 mice. We further subjected the mice to the novel object recognition (NOR) test, a paradigm of hippocampus‐dependent short‐term memory.^55^ Although the total exploration time around novel and familiar subjects was similar between TMP269 and vehicle in APP/PS1 mice (Figure 6F,G), the DI was significantly enhanced in TMP269‐treated APP/PS1 mice, compared to that in vehicle‐treated APP/PS1 mice (Figure 6F,H), indicating that HDAC9 inhibition attenuates the deficits in hippocampal short‐term memory of APP/PS1 mice.

Lastly, we examined whether HDAC9 inhibition rescues impaired fear memory of APP/PS1 mice. The mice were subjected to training (i.e., pairing with the US—electric shock and CS—contextual box). One day later, freezing or fear memory was tested when mice were returned to the contextual box (Figure 6I). Two groups of mice were subjected to contextual fear training and tests (Figure 6J). TMP269‐treated APP/PS1 mice showed increased frequency of freezing, compared to vehicle‐treated APP/PS1 mice, indicating that HDAC9 inhibition rescues impaired fear memory of APP/PS1 mice (Figure 6K). Taken together, these results indicate that HDAC9 inhibition ameliorates hippocampus‐dependent memory impairment in APP/PS1 mice.

## 4 DISCUSSION

The present study reveals a novel regulatory mechanism underlying the posttranslational modification of CaM in AD. In this study, we provide evidence for that HDAC9 is a novel deacetylase of CaM. We further demonstrate that abnormally upregulation of HDAC9 contributes to cognitive deficits of AD via causing hypoacetylation of CaM, highlighting a pathological role of HDAC9‐mediated CaM deacetylation in AD pathology.

CaM, as a calcium sensor, regulates learning and memory by activating kinases such as CaMKIIα.^28^ In previous studies, we demonstrated that SRC3‐mediated CaM acetylation was important for CaMKIIα activity and hippocampus‐dependent memory in mice.^31^, ^45^, ^46^ Here, we showed that HDAC9‐mediated CaM deacetylation and CaMKIIα activity are severely abnormal in AD mouse brain. In addition to CaMKIIα, CaM has many other target proteins, several of which have been implicated in synaptic plasticity, neurotransmission or neuronal excitability.^21^ For example, CaM regulates NR1 subunit,^22^ L‐type calcium channel,^23^ cGMP‐gated cation channel^56^ and other ion channels.^24^ Future work is warranted to investigate whether HDAC9‐mediated CaM deacetylation alters the function of these proteins in AD.

To screen which HDAC participate in the deacetylation of CaM, we performed shRNA screen against HDAC. Since the class I HDAC, HDAC1, 2, and 3 were mainly distributed in the nucleus, the class II HDAC were expressed in the nucleus and cytoplasm, and CaM is a cytoplasmic protein, so we chose class II HDAC4,6‐9 with the same distribution as CaM. We performed unbiased screen to identify HDAC9 and HDAC6 as the deacetylase for CaM in HEK293 cells. Since HDAC9 deacetylates CaM more strongly than HDAC6 in HEK293 cells, we used HDAC9 for further verification and functional experiments. However, we did not exclude that HDAC6‐mediated CaM deacetylation is involved in AD pathology. HDAC9 is known to be expressed in the brain, however, its function in the brain is largely unknown. *Hdac9* mRNA is enriched in the pyramidal neurons of the hippocampus from Allen Brain Institute (https://mouse.brain‐map.org/gene/show/55058), in agreement with a previous report.^57^ Here we demonstrated that HDAC9 is the main deacetylase for CaM. Remarkably, site‐specifically acetylated‐CaM could be deacetylated by HDAC9 purified from HEK293 cells. These results suggested that CaM is a direct substrate of HDAC9. This is the first time that the deacetylase of CaM has been identified as HDAC9, and CaM is also the first nonhistone deacetylation substrate of HDAC9.

In AD patients and 5‐ or 7‐month‐old AD mice, K22 and K95‐CaM acetylation decreased significantly (Figures 1, 2 and 4). However, 7‐month‐old AD mice have not yet emerged massive accumulation of amyloid‐β plaques and tau tangles, dendritic spines loss, neuronal cell death and brain atrophy.^35^, ^36^, ^37^, ^38^, ^39^, ^40^, ^41^ These results highlight a casual role of CaM acetylation in AD pathogenesis, suggesting that abnormally reduced plasma K22 and K95‐CaM acetylation may be a potential new early diagnostic marker for AD. The ~1% stoichiometry level of K22 and K95‐CaM acetylation in the total lysates of forebrain was similar to those of bona fide acetylated proteins in mammalian cells.^58^ The calcium elevation during learning is localized to stimulated spines, or a region called calcium nanodomain which is near the inner mouth of postsynaptic NMDA receptor.^28^ One could speculate that the stoichiometry level of CaM acetylation in the stimulated spines or calcium nanodomain might be much higher than that in the total lysates. Future studies are warranted to generate new tools to study CaM acetylation in the stimulated spines or calcium nanodomain in AD.

HDAC9 is abnormally increased in 7‐month‐old APP/PS1 mouse brain (Figure 6A,B). However, 12‐month‐old AD mice have emerged obvious dendritic spines loss and neuronal cell death,^35^, ^36^, ^37^, ^38^, ^39^, ^40^, ^41^ these may be the reasons why HADC9 is reduced in the brains of 12‐month‐old AD mice.^29^ These results suggest that only intervention of HDAC9 in the early clinical stage of AD could play a therapeutic role in the future. The abnormal increase of HDAC9 may be related to the APP/PS1 transgenic mice, which express a chimeric mouse/human Swedish mutant APP (APPswe) and a mutant human presenilin 1 (PS1). Recent study reported that HDAC9 is a risk gene for AD,^59^ this further proved that HDAC9 is involved in AD pathogenesis. Future work is warranted to investigate why APP/PS1 transgenic mice cause the abnormal increase of HDAC9, and whether other AD mouse models have similar changes.

AD is a chronic neurodegenerative disease characterized pathologically by the accumulation of amyloid‐β plaques and tau tangles, which leads to neuronal cell death, cognitive impairment. This paper mainly studies the role of HDAC9‐mediated CaM deacetylation in memory impairment of AD mice, and whether HDAC9‐mediated CaM deacetylation is involved in accumulation of amyloid‐β plaques and tau tangles, neuronal cell death and dendritic spines loss, to be systematically studied in the future. One thing to note, since the microtubule binding protein tau promotes the assembly and stabilization of microtubules,^60^ accumulation of tau tangles leads to structural changes in the brain. However, CaM is a functional regulatory protein in synaptic plasticity,^61^ abnormal reduction in CaM acetylation may be more related to brain dysfunction in AD early stage, this is consistent with the previously reported calcium dysregulation in AD early stage.^62^, ^63^, ^64^, ^65^, ^66^

## 5 CONCLUSION

In general, our results reported HDAC9‐mediated CaM deacetylation induced memory impairment in AD, and discovered that abnormally reduced plasma CaM acetylation may be a new early diagnostic marker for AD, and indicated that HDAC9 or CaM acetylation may become potential therapeutic targets for AD.

## AUTHOR CONTRIBUTIONS

H‐L.Z. performed experiments, analyzed the data, prepared the figures, and wrote the manuscript. S.H. and P.Y. performed the experiments. H‐C.L. collected human blood samples. Q‐H.M. edited the manuscript. G‐Y.X. and D‐M.Y. supervised the work. G‐Y.X., D‐M.Y. and H‐L.Z. designed the study and finalized the paper. All the authors have approved the paper.

## CONFLICT OF INTEREST STATEMENT

The authors declare no competing financial interests.

## Supporting information


Figures S1–S5.

## Data Availability

The data that support the findings of this study are available from the corresponding author upon reasonable request.
