# Supplementary material for: HDAC9‐mediated calmodulin deacetylation induces memory impairment in Alzheimer's disease
Source: CNS Neurosci Ther. 2024 Feb 7;30(2):e14573. doi: 10.1111/cns.14573 (PMC10850929; doi:10.1111/cns.14573)

Figure S1. Full unedited blots for Figure 1.

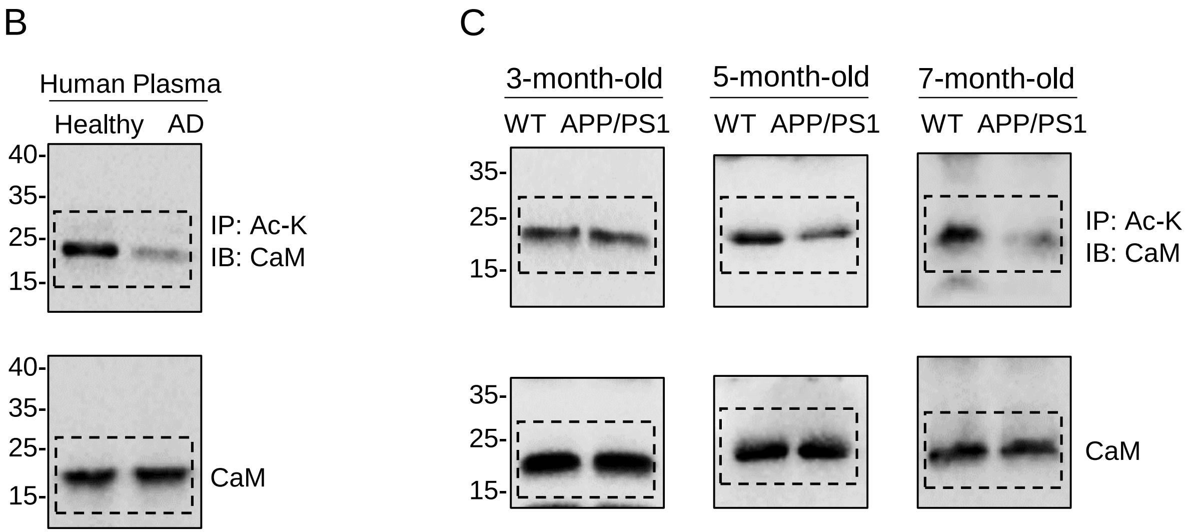

Figure S2. Full unedited blots for Figure 2.

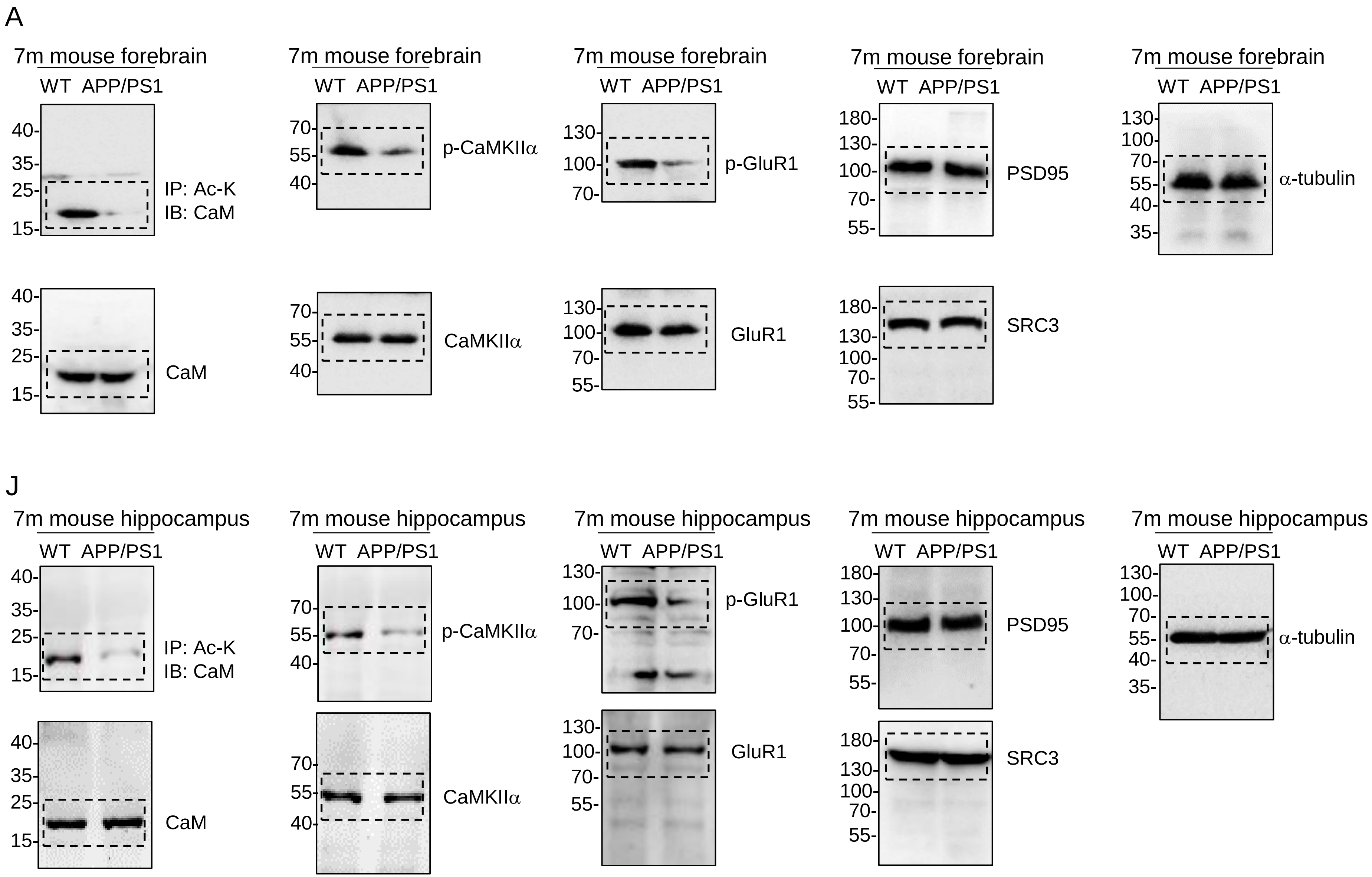

Figure S3. Full unedited blots for Figure 3.

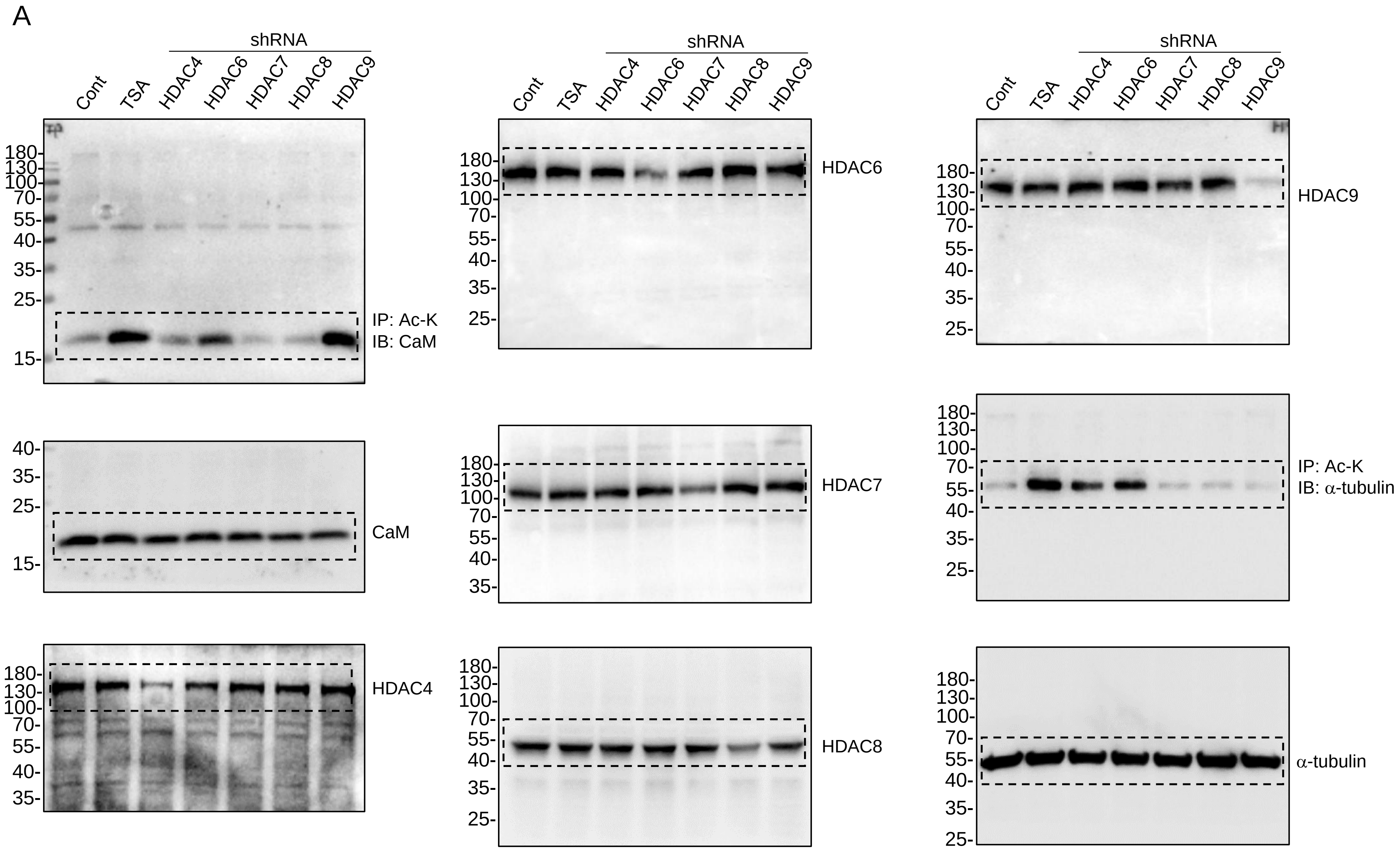

Figure S4. Full unedited blots for Figure 4.

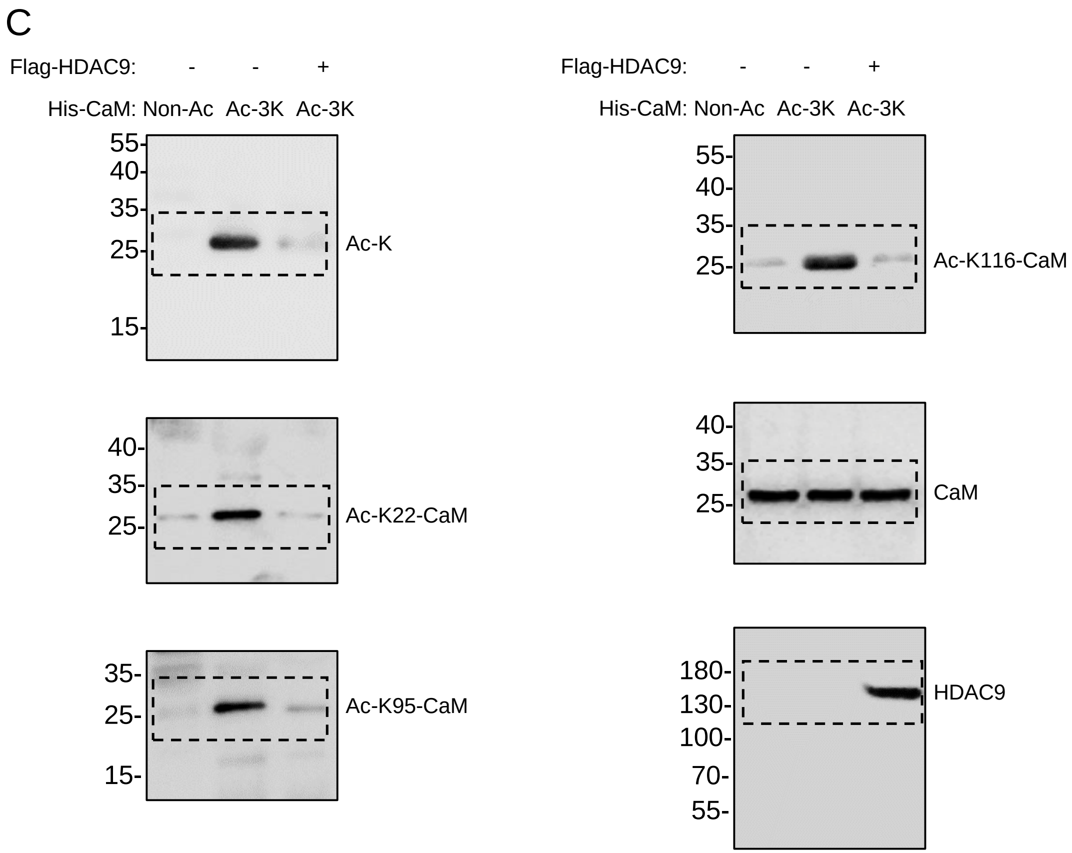

Figure S5. Full unedited blots for Figure 5.

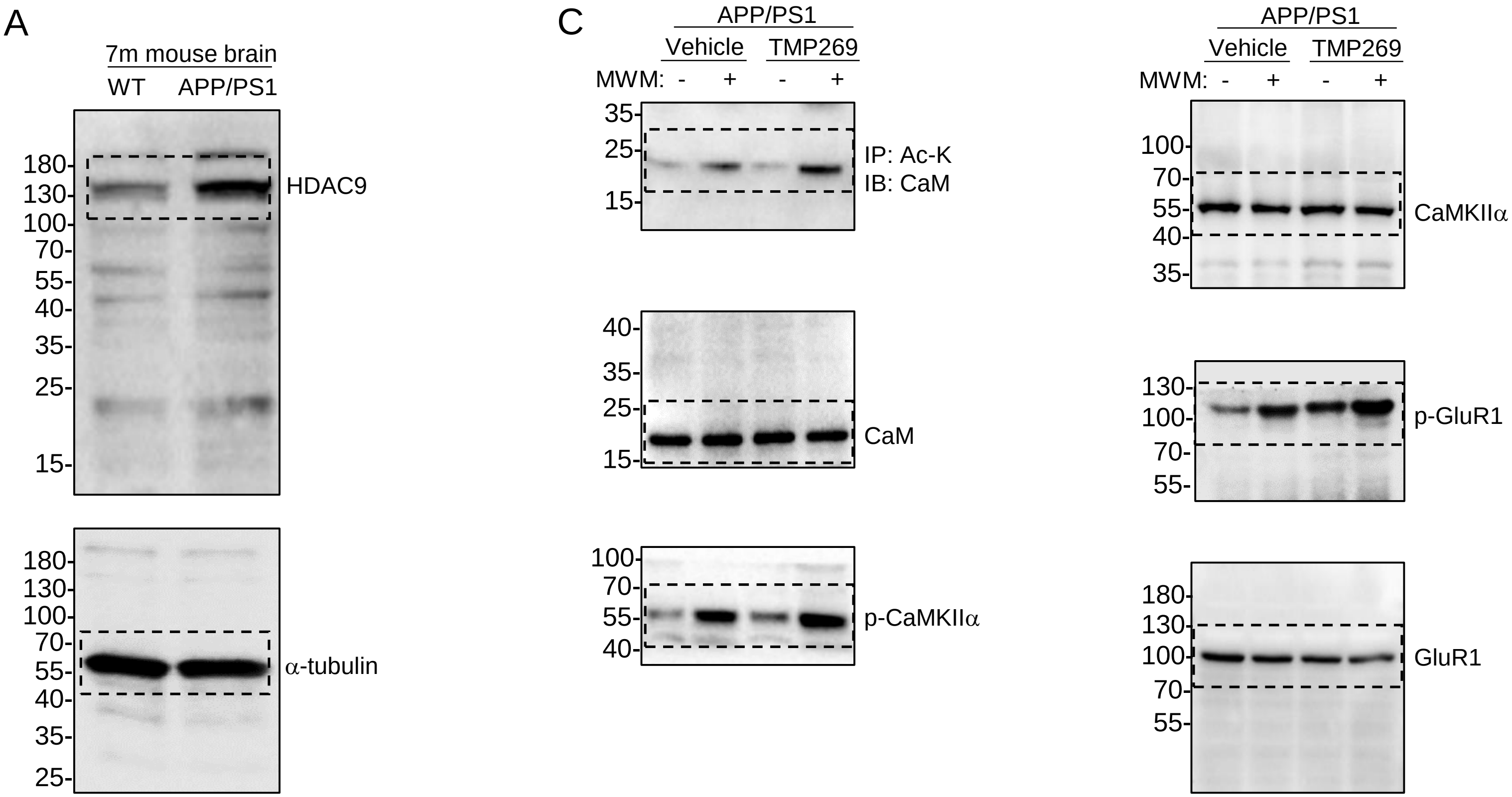

Supplement: Supplementary file 1 — Figures S1–S5. [file CNS-30-e14573-s001.pdf]
